# Stakeholder perspectives on skills required for health technology developers: a qualitative study in Thailand

**DOI:** 10.3389/fdgth.2025.1578782

**Published:** 2025-07-18

**Authors:** Sranya Phaisawang, Thanyaphong Na Nakorn, Prasert Trivijitsilp, Anan Srikiatkhachorn

**Affiliations:** Faculty of Medicine, King Mongkut’s Institute of Technology Ladkrabang, Bangkok, Thailand

**Keywords:** health technology, Thailand's digital health ecosystem, qualitative study, skills for health technology, health technology development

## Abstract

**Background:**

Throughout history, medical education has developed in response to societal changes and advances in biological research and technology. Health technology, encompassing devices, medicines, vaccines, and digital health systems, is transforming healthcare with increased effectiveness and efficiency. Thailand, a popular medical tourism destination, intends to shift its focus to high-quality healthcare services and advanced technologies for long-term economic sustainability. This study identifies necessary skills for health technology developers to help create a technology-driven healthcare ecosystem and prepare human capital in the field.

**Methods:**

In this qualitative study, in-depth interviews with diverse stakeholders in health and health technology industries were conducted to investigate the role of health technology in future healthcare and the skills required for health technology developers. Qualitative Content Analysis was carried out. Participants included national health policy makers, university presidents, hospital directors, and health technology company administrators. The study utilized the electronic Delphi method for ranking skills through multiple interview rounds, ensuring thorough evaluation of significant topics.

**Results:**

This study involved interviews with sixteen stakeholders in health technology, focusing on its importance and impact on future healthcare. Participants discussed three major areas of technology: molecular technologies, biomedical engineering technologies, and health information technologies. Delocalization, personalization, and digitalization are key components of healthcare transformation. The challenges and skills needed for health technology developers were categorized into four domains including, Health Science, Health Technology, Product Development & Design and Marketing & Entrepreneurship.

**Conclusion:**

Our study revealed the significance of technology in healthcare transformation. We identified four skill categories that health technology developers must possess. (1) Health Science, (2) Health Technology, (3) Product Development & Design, and (4) Marketing & Entrepreneurship were among these domains. A systematic strategy for developing these skills is a crucial success factor in human capital preparation for future technology-driven healthcare.

## Introduction

Digital health is the incorporation of digital technologies in health, healthcare, living and society to improve the efficiency of healthcare provision and to make medicine more accurate as well as tailored (1, 2). It entails several technologies and platforms such as health information technology, mobile health (mHealth), wearable devices, telehealth and big data analytics in healthcare. At its heart, digital health seeks to use Information and Communication Technologies (ICTs) to address wellness and health issues, thereby changing how healthcare is accessed, delivered and managed.

Digital health innovations are revolutionizing healthcare and requiring a change in medical education. Future healthcare professionals need to be trained in these new paradigms as the field shifts toward personalized, predictive, participatory and preventive methods. Students must comprehend the uses and constraints of artificial intelligence (AI) and machine learning (ML) which enhance diagnosis and treatment planning. Medical education needs to change to better prepare students for the new environment as digital health advances to address global health issues.

Throughout its history, medical education has been continuously evolving from pure apprenticeship during the medieval period to current formal and highly structured biomedical science-based education. This transformation was a response to changes in various societal attributes such as culture, expectation, biomedical and technological advancement. Current medical education comprises basic sciences and clinical sciences, which has been proven very effective in producing scientifically grounded and clinically skilled physicians (3–5). However, modern factors force transformation of future medical practice, requiring health professionals to develop new skills.

Advancement in health technology is one major force that has progressively driven healthcare transformation (6, 7). According to the World Health Organization (WHO), health technology is defined as the “application of organized knowledge and skills in the form of devices, medicines, vaccines, procedures, and systems developed to solve a health problem and improve quality of lives” (8). Technological advances and new innovations are leading to a new dimension of health interventions, either diagnostic or therapeutic, which are more effective and more efficient for disease diagnosis via smart healthcare including innovation, technologies and applications (9–11). Access to high quality, affordable, and appropriate health technologies is indispensable to advance universal health coverage, address health emergencies, and promote healthier populations. As a result, medical practice becomes more and more reliant on technologies of the future.

Health technology also includes computer-supported information systems and organizational systems that are used in the healthcare industry. Clinical informatics and health information technology are integral components of health system science, a discipline considered to be the third component of medical education, alongside the other two pillars of basic and clinical sciences (12, 13). Health technology includes digital health, which has the potential to enhance numerous aspects of healthcare including diagnosis and treatment, continuity of care, remote patient management through telemedicine and partnership with individuals to encourage self-management (14). Nowadays, artificial intelligence (AI), and machine learning, (ML), advances are growing quickly in many sectors, especially in healthcare. AI applications could revolutionize physician workflows and patient care from replacing administrative tasks with autonomous assistance to augmenting expertise (15).

Digital healthcare and medical tourism are becoming more popular around the world, especially in Asia and Southeast Asia for its high standard of care, accessibility and cost effectiveness (16). Thailand is an upper middle-income country which has a good reputation in medical services. This developing nation has 60 hospitals that meet Joint Commission International (17) accreditation standards, the highest number in ASEAN and the fourth-most in the world, making the country ready to profit from medical tourism.

According to the Medical Tourism Index (MTI) conducted by the International Healthcare Research Center (IHRC), Thailand was ranked 17th as the most popular medical tourism destination in 2020 with the MTI score of 66.83 (18). In terms of quality and services, Thailand is ranked 15th out of 46 destinations. Thailand is shifting its focus from the quantity of medical tourists to more of a quality of healthcare service aspect, to achieve a sustainable medical tourism sector with high quality, attractive technical skills, and advanced technologies, which will be able to support the overall economy in the long term. One key factor to boost Thailand as a world-class medical hub is the promotion of a dynamic health technology ecosystem that supports the transformation of the organization-centered healthcare model into a patient-centered model. However, there are some obstacles and limitations that need to be addressed.

Despite growing investment in health technology development, there is limited research identifying essential skills required for health technology developers from stakeholder perspectives for curriculum development, professional training, and workforce preparation in technology-driven healthcare systems. The successful development of Thailand's health technology ecosystem requires systematic identification of necessary skills and competencies.

The objectives of this study are to explore stakeholder perspectives on the role of health technology in healthcare transformation and to identify the essential skills required for health technology developers to successfully design, develop, and implement health technology solutions. The results of the study are beneficial both in terms of the development of appropriate ecosystem for health technology and preparation of human capital, both medical and non-medical, for future technology-driven healthcare.

## Materials and methods

### Expert committee selection

In this qualitative study, we used the in-depth interview method (19). Interview participants included industry experts ranging from C-level executives and higher in related government, medical education and healthcare sectors. Table 1 shows the four different areas of expertise in health and health technology related industries. Firstly, decision makers in the highest governmental levels from the Former Permanent Secretary of the Ministry of Higher Education, Science, Research and Innovation and Chairman of the Medical Device Committee of the National Research Council of Thailand were identified as knowledgeable experts that represent national health technology policies that drive Thailand's future. University presidents, from both public and private institutions, were also invited to participate in the interviews as this group of educational administrators offered insight on the educational outlook and outcomes of teaching and course development towards a more medical and technologically advanced approach. Purposive sampling was used in our recruitment process to guarantee a knowledgeable and diverse panel of experts. A minimum of ten years of experience in a senior role in the domains of health technology, healthcare administration, health policy or related fields was required for inclusion. Established professionals who had active participation in policy making, implementation or development of health technology, with readiness to take part in several rounds of the Delphi process were approached. Through academic journals, industry associations, recommendations and professional networks, we were able to find potential participants to join the study. Following email correspondence, a formal invitation letter outlining the goals and methods of the study was sent out to each prospective participant.

**Table 1:** Population categories of sample.

Category	Level of respondents
National health policy makers	Former Permanent Secretary of the Ministry of Higher Education, Science, Research, and Innovation
	Chairman of the National Research Council of Thailand
University administration	Public University President
	Private University President
Public and private hospital personnel	Public Hospital President
	Private Hospital President
Health technology personnel	Health Technology Company Administrator
	Health Technology Company Developer

Private and public hospital presidents represented a part of the nation's medical technology ecosystem as both hospital types are active testing grounds for the integration of technology-driven healthcare services with its end users identified as high-potential customer base for medical technological innovations. To ensure that all the major stakeholders in the medical technology ecosystem were represented, health technology developers were included to offer insight, from the R&D and builder's perspective, on the health technology innovation process. Each of the participants were selected for their extensive expertise (over 10 years) in executive positions in medical, health technology, educational administration, and health technology development.

### Qualitative interviews

A qualitative analysis of the 16 semi-structured stakeholder interviews was undertaken using the Qualitative Content Analysis Theory. The study description and results were summarized in accordance with the Consolidated Criteria for Reporting Qualitative Research checklist. Participants were invited to participate in the study by personal contact, followed by an official letter of invitation via email. Since no exact definition specifying between a small and large sample exists, and because there is no disagreement or argument against the recommended size of a Delphi study panel, this situation-specific qualitative analysis recruited convenience samples of major stakeholder participants and was done so based on availability of experts which were comprised of national health policy makers, university presidents, hospital directors, health technology company administrators and health technology developers as previously mentioned in Table 1 (20, 21). Participants were excluded if they were not available to participate in the initial in-depth interviews and discontinued if they were unavailable to participate in all three rounds of the e-Delphi interviews. Initially, 18 experts were invited to participate in the study, however, two were excluded due to scheduling conflicts, leaving a total of 16 expert participants who were included in the study. Written consent was obtained from all participants. Included participants who did not complete all three rounds of the e-Delphi interviews are considered withdrawn from the study. Withdrawal was voluntary and any information gathered up to the date of withdrawal was retained.

Semi-structured interviews were conducted with 16 experts to gather in-depth insights on health technology and required skills for developers. To ensure the trustworthiness of this qualitative phase, we implemented several strategies. First, we used member checking which involved returning interview transcripts to participants for approval and explanation. This allowed them to attest to the veracity of their claims and offer any further context. Second, in order to reduce individual bias in interpretation, we used peer debriefing in which two researchers (SP and AS) independently analyzed the interview data and then compared and discussed their findings to reach a consensus. Thirdly, we kept a thorough record of all the research activities including raw data analysis notes and introspective writings. This ensured transparency and the possibility of future replications. Finally, we used triangulation—using information from several sources such as literature reviews, expert panel discussions, and interviews—to strengthen the validity of our conclusions. With this thorough approach to trustworthiness, the goal was to generate solid and trustworthy qualitative data that would serve as the basis for the ensuing Delphi rounds.

The interviews were semi-structured and a topic list was prepared in advance and piloted. The interview topic guide included (1) a section relating to the importance of health technology and its impacts on future healthcare; (2) necessity of health technology developers and their role in healthcare transformation; and (3) skills required for being a health technology developer. Interviews were undertaken online and were recorded. Interviews were conducted until saturation was reached, as demonstrated by the absence of new themes emerging from the analysis. Interviews were independently analyzed by two researchers (SP and AS).

Information regarding the skills required for being a health technology developer were extracted from the interview transcripts. Related required skills were categorized and skills in each category were listed alphabetically. In the Delphi ranking process, participants ranked skills within each domain in order of importance, with 1 representing the highest priority/most important skill and higher numbers representing lower priority. The Health Science domain contained 7 skills (ranked 1–7), Health Technology contained 11 skills (ranked 1–11), Product Development & Design contained 6 skills (ranked 1–6), and Marketing & Entrepreneurship contained 9 skills (ranked 1–9). A list of required skills was sent back to the interviewees for ranking via electronic mail. The final rank was obtained via three rounds of the e-Delphi method (see details in Supplementary Appendix A). The e-Delphi method with multiple interview rounds was deployed (22). All rounds of the interviews were undertaken by AS. Information regarding the study was sent to the interview participants via email prior to each interview. Replies were then collected, and themes were identified and evaluated for each round until the major themes were identified.

Ethics exemption was granted by the Institutional Review Board, King Mongkut's Institute of Technology Ladkrabang.

## Results

Sixteen stakeholders comprising health technology developers (7), national health policy makers (2), university presidents (2), hospital directors (2) and health technology company administrators (3) were interviewed between August and December 2021. The recruitment rate from invitation was 80%. All interviews were undertaken via online platform. Each round of interviews had a median length of 22 min (range 12–35 min).

### Importance of health technology and its impacts on future healthcare

All stakeholders agreed that health technologies strongly impacted healthcare transformation. Technologies which will drive healthcare transformation included artificial intelligence, data analytics, telehealth, internet of thing (IoT), sensors, point-of-care diagnostics, and personalized/precision medicine. These technologies can be classified into three main categories.

1. Molecular technologies: The main technology in this category is high throughput technologies. These techniques enable physicians to characterize the biological markers (biomarkers), including deoxyribonucleic acid (DNA), ribonucleic acid (RNA) or protein, by massively parallel sequencing. With the increase in technological capacity and decrease in cost, routine sequencing tests can become more available and accessible to better guide patient management, including disease prediction, prevention, and treatment decisions. This will facilitate the development of personalized medicine.
2. Biomedical engineering technologies: This category includes medical sensors, surgical robots, assistive technologies, biomaterials, three-dimensional printing technologies, microdevice development, point-of-care diagnostics, etc. These technologies will increase the performance of health services. For example, surgical robots can help access organs that are difficult to approach, provide more precise and less invasive operations. Advancement in medical sensors, microdevice development and point-of-care diagnostics will enable patients to be more engaged with the healthcare system.
3. Health information technologies: Information technologies has a strong impact on healthcare in various aspects. Development of online databases is the core infrastructure of bioinformatics which enables complex genome and protein analysis. Development in medical artificial intelligence can enhance efficiencies of medical imaging technologies with accurate image analysis capabilities. It also helps in the design of personalized treatment for patients based on their medical history. Personal health data from various sources such as personal tracking devices, medical records, laboratories, etc. can be uploaded and retrieved for analyses. Blockchain technology will make personal health records more accessible with heightened security. An online health platform can link physicians with their patients. Eventually, healthcare will undergo digital transformation in which digital technology is completely integrated into all areas of health services and will fundamentally change how healthcare services are operated and delivered.

Based on the three technology categories mentioned above, healthcare will be transformed with three key aspects including delocalization, personalization, and digitalization. The center of healthcare will move from medical institutes such as hospitals to more personalized environments such as the patient's home. Healthcare can be delivered everywhere to serve patients in remote locations as well as decrease expenditures and costs while increasing efficiency and convenience. Advances in high throughput technologies and health informatics will foster personalized medicine for more responsive healthcare management for many different levels of patients ranging from the elderly to the underserved populations. Healthcare will see a shift from the application of statistically certified paradigms (such as in evidence-based medicine) to more personalized protocols that consider the patient's (genomic) specificity.

### Necessity of health technology developer and its role in healthcare transformation

All stakeholders appreciated the importance of health technology developer in the process of healthcare transformation. The Policy Makers group described a shortage of human capital in the health technology industry at the national level. They also pointed out that, in order to improve the Thai health technology industry, three main areas require development. These include: (1) establishment of national standards; (2) a market for health technology and investment for sustainable growth; and (3) eco-system for the medical device industry that includes technology and human resources. This group emphasized the necessity of an urgent upscaling of domestic expertise and technology to match that of the international level in addition to long-term research funding in the development of human resources and technology related trends, both current and future, in the medical device industry.

The University Administrator group mentioned their roles in producing health technology developers. They described the programs and courses in their universities that are involved in the process. However, there is no comprehensive program that covers all skills required for being a competent health technology developer.

Health Technology Company Administrators and Health Technology Developer groups described limitations in the infrastructure for health technology development. Lack of a national center for health technology certification was a main obstacle and barrier to success. This lack of a national certification center for health technology prohibits the process of launching newly developed technologies in the market. These experts also highlighted that another problem is that local health technology companies are less competitive when compared to large international companies. Since purchase decisions for health technologies depends on trust, local companies in Thailand face adversities in branding therefore, international companies with long-standing track records are more trustworthy and have more advantage in terms of market competition.

### Skills required for being a health technology developer

All stakeholders described the skills required for being a health technology developer. The skills were categorized into four domains including (1) Health Science, (2) Health Technology, (3) Product Development & Design, and (4) Marketing & Entrepreneurship. After three rounds of the Delphi method, skills in each domain were ranked (Tables 2, 3). Table 4 shows the final list of top skills required for health technology developers.

**Table 2:** Skills required for health technology developer ranked by stakeholder category.

Skill	Policy makers	Univ. admin	Hosp. admin	Health tech ad	Health tech dev
Health science					
1. Precision and personalized medicine	2	2	2	1	1
2. Scientific method	1	1	1	4	2
3. Human cognitive functions	3	3	5	3	3
4. Diagnostic laboratory techniques	4	4	2	2	5
5. Medical imaging techniques	7	5	4	7	4
6. Molecular mechanisms of drug action	5	6	6	5	6
7. Molecular pathobiology	5	7	7	6	7
Health technology					
1. Artificial intelligence for healthcare	1	1	1	2	1
2. Data sciences in healthcare	2	2	2	1	4
3. Bioinformatics	3	6	6	3	2
4. Signal processing and data analytics	6	5	3	4	2
5. Virtual and augmented realities	3	4	9	7	9
6. Computer programming	10	3	5	9	5
7. Software engineering	5	7	4	11	8
8. Biosensors	6	8	11	8	5
9. Healthcare robotics	8	9	7	5	10
10. Biomaterial	11	11	8	5	5
11. Hospital information system	9	10	10	10	11
Skill	Policy makers	Univ. admin	Hosp. admin	Health tech ad	Health tech dev
Product development and design					
1. Design thinking	1	1	1	1	2
2. Clinical innovation and design	2	2	2	3	1
3. User experience and user interface	4	3	4	4	3
4. Technology and ethics	3	5	6	2	4
5. Sociocultural perspectives to innovative technologies	5	6	3	6	5
6. Universal design	6	4	5	5	6
Marketing and entrepreneurship					
1. E-market and digital healthcare	1	1	1	5	2
2. Medical device approval and certification	5	3	5	1	1
3. Healthcare industry and market	3	5	2	4	3
4. Healthcare market research	4	6	6	3	4
5. Medical device business strategy	2	7	4	6	5
6. Innovation and intellectual property	9	2	6	2	6
7. Entrepreneurship in technology and health	8	4	3	8	7
8. Healthcare financing	6	9	8	6	9
9. Device branding for commercialization	7	8	9	9	8

Skills were ranked after three rounds of the Delphi method with 1 representing highest priority and higher numbers representing lower priority within each domain.

**Table 3:** Top three skills in each domain as ranked by each stakeholder category.

Domain	Policy makers	University presidents	Hospital directors	Health tech administrators	Health tech developers
Health science	1. Scientific method	1. Scientific method	1. Scientific method	1. Precision and personalized medicine	1. Precision and personalized medicine
	2. Precision and personalized medicine	2. Precision and personalized medicine	2. Precision and personalized medicine	2. Diagnostic laboratory techniques	2. Scientific method
	3. Human cognitive functions	3. Human cognitive functions	3. Diagnostic laboratory techniques	3. Human cognitive functions	3. Human cognitive functions
Health technology	1. Artificial intelligence for healthcare	1. Artificial intelligence for healthcare	1. Artificial intelligence for healthcare	1. Data sciences in healthcare	1. Artificial intelligence for healthcare
	2. Data sciences in healthcare	2. Data sciences in healthcare	2. Data sciences in healthcare	2. Artificial intelligence for healthcare	2. Bioinformatics
	3. Bioinformatics	3. Computer programming	3. Signal processing and data analytics	3. Bioinformatics	3. Signal processing and data analytics
Product development & design	1. Design thinking	1. Design thinking	1. Design thinking	1. Design thinking	1. Clinical innovation and design
	2. Clinical innovation and design	2. Clinical innovation and design	2. Clinical innovation and design	2. Technology and ethics	2. Design thinking
	3. Technology and ethics	3. User experience and user interface	3. Sociocultural perspectives to innovative technologies	3. Clinical innovation and design	3. User experience and user interface
Marketing & entrepreneurship	1. E-market and digital healthcare	1. E-market and digital healthcare	1. E-market and digital healthcare	1. Medical device approval and certification	1. Medical device approval and certification
	2. Medical device business strategy	2. Innovation and intellectual property	2. Healthcare industry and market	2. Innovation and intellectual property	2. E-market and digital healthcare
	3. Healthcare industry and market	3. Medical device approval and certification	3. Entrepreneurship in technology and health	3. Healthcare market research	3. Healthcare industry and market

**Table 4:** Top skills required for health technology developers.

Domain	Knowledge & skill
Health Science	– Scientific method – Precision and personalized medicine – Human cognitive functions – Diagnostic laboratory techniques
Health technology	– Artificial intelligence for healthcare – Data sciences in healthcare – Bioinformatics – Signal processing and data analytics
Product development & design	– Design thinking – Clinical innovation and design – User experience and user interface – Technology and ethics
Marketing & entrepreneurship	– E-market and digital healthcare – Healthcare industry and market – Medical device approval and certification – Innovation and intellectual property

Kendall's concordance coefficient is also used to determine the level of agreement between experts. Kendall's coefficient W ranges between 0 and 1 in general; the larger the value, the better the degree of coordination among experts. Kendall's test result's statistical significance indicates agreement among experts. The expert coordination coefficients as shown in Table 5, were statistically significant (*P* < 0.01) in all dimensions in the third Delphi round. This indicates that all experts have a tendency to agree (23–26).

**Table 5:** Kendall's W concordance coefficient test.

Scoring criteria	Round 3			
	*W*	*χ* ^2^	*df*	*P*
Health science	0.771	23.118	6	0.001
Health technology	0.658	32.919	10	0.000
Product development and design	0.739	18.486	5	0.002
Marketing and entrepreneurship	0.562	22.495	8	0.004

Concerning the Health Science domain, top skills required were scientific method, precision and personalized medicine, human cognitive functions, and diagnostic laboratory techniques. The scientific method is an indispensable foundation of rigorous, systematic research that supports evidence-based treatments and enhances our understanding of disease. Precision and personalized medicine takes this concept one step further by tailoring treatments and interventions based on each patient's genetic makeup, lifestyle choices and medical history. Furthermore, cognitive functions—mental processes that support decision-making, problem solving and communication skills among others—are vital tools used by healthcare providers when diagnosing and treating their patients. Diagnostic laboratory techniques are essential in the diagnosis and monitoring of various medical conditions ranging from cancer to infectious diseases. These techniques require many skills including sample collection, analysis and interpretation, which are essential elements to providing timely diagnosis and effective treatment options. It is noteworthy that hospital directors and administrators of health technology placed strong emphasis on diagnostic laboratory techniques, reflecting their significance in healthcare delivery as well as laboratory professionals' roles. This statement underscores the complexity of Health Science as a field requiring varied skill sets for success.

The processing of digital information has been identified by the Health Technology domain as the most important skill. The fact that the top four skills are all directly related to data processing confirms this finding. These four skills include: artificial intelligence in healthcare, data science in healthcare, bioinformatics & signal processing, and analytics. Digital data processing is crucial to the Health Technology domain, as it allows for valuable insights to be extracted and more efficient and effective healthcare solutions developed. The rapid development of technology, and the availability of more data is driving the demand for digital data processing experts in the healthcare field.

The Hospital Directors Group has identified four skills as essential in the area of Product Development & Design. The skills include design thinking, clinical design & innovation, user experience and user interface, technology & ethical considerations. These skills are essential for the design and development of innovative healthcare solutions. They also ensure that products are ethically sound. It is also important to note the Hospital Directors' group expressed concern about the sociocultural aspects of innovative technologies. They are aware of the possible cultural differences that can exist when using health technology. These concerns highlight the importance of considering the sociocultural influences that can impact the development and design of healthcare products. It is important to make sure that these products are accessible and inclusive to all groups, regardless of cultural background or socioeconomic standing. Incorporating sociocultural perspectives in the product development process allows healthcare organizations to create solutions that are both effective and culturally appropriate.

There are several skills that are crucial to success in the Marketing & Business domain of the Healthcare Industry. These skills include digital health and E-market, the healthcare industry and market as well as medical device certification and approval. These skills are essential to the development and marketing of successful healthcare products and service. It is important to note, however, that the Policy Maker Group has highlighted the importance of wider areas such as the medical device business strategies. This shows the importance of a holistic approach to marketing and entrepreneurship within the healthcare industry. It involves understanding not only the specific skills needed but also the wider strategies and tactics that are necessary for success.

On the other hand, technology developers, both at the administrative and developer level, have shown a greater concern for practical issues such as the approval and certification of medical devices. This is due to the fact that it is important to have a thorough understanding of the processes and requirements involved in gaining approval for devices. These findings, taken together, demonstrate the multifaceted nature and complexity of the Marketing & Entrepreneurship in Healthcare domain, which requires a variety of skills and expertise. Understanding the needs and concerns specific to different stakeholder groups allows healthcare organizations to develop marketing and entrepreneurship plans that are tailored for their needs and goals.

## Discussion

In this study, we reported the perceptions of four groups of stakeholders about health technology. Our study identified three main health technology categories that have strong impact on healthcare namely molecular technologies, biomedical engineering technologies and health information technologies. After three rounds of the Delphi method, four domains of skills that are required for health technology developers were identified. These domains included (1) Health Science, (2) Health Technology, (3) Product Development & Design, and (4) Marketing & Entrepreneurship.

Concerning the Health Science domain, scientific method was ranked as the most important skill. This reflects that scientific method is the foundation for further technology development and innovation (27) and underscores the pliability of the scientific method, which allows for modifications based on novel data. This methodical approach, employed to evaluate hypotheses and formulate theories, has considerably propelled fields such as medicine, physics, and chemistry (28). Moraes (29) also highlights the role of the scientific method in developing validated scientific information for decision making in healthcare. The related skills in this area include (1) making an observation; (2) asking a question; (3) forming a hypothesis; (4) making a prediction based on the hypothesis; (5) testing the prediction; and (6) using the results to make new hypotheses or predictions. Universities as well as medical schools should design their teaching/learning activities to foster the development of these core skills. Project-based learning, an educational approach in which students explore real-world problems through individual and group projects, is one good option that can expose medical technology developers to a setting conducive of technology development and innovation early on and foster skill development. Thomas (30) set five criteria for project-based learning which included (1) projects should be central to the curriculum; (2) focused on problems that drive the students to struggle with major concept; (3) involve the students in constructivist investigation; (4) student-driven; and (5) realistic.

The other highest priority in Health Science domain was precision and personalized medicine. This discipline requires an integration of knowledge and skills in various areas, such as high-throughput technology, bioinformatics, data science, etc. Information regarding this broad discipline should be integrated in medical curriculum in order to prepare medical graduates for future medical practice in a technology-driven healthcare landscape.

Our study highlighted the importance of digital data processing. Top skills in this domain as identified by our interviewees were artificial intelligence, data sciences in healthcare, bioinformatics & signal processing & data analytics. AI and precision medicine hold immense promise to revolutionize healthcare by identifying patient phenotypes, and providing customized diagnosis and prognostication through artificial intelligence (31). These findings supported the concept of digital transformation of healthcare (32–34). Digital transformation of healthcare requires not only the development of electronic health data and platform, but also other components. According to Pousttchi et al. (35), digital transformation of healthcare requires the integration of digital technologies in conjunction with (1) comprehensive use of sensors and actors including audio and video recordings; (2) use of mobile communication technologies for networking and automated communication with very low latency (Internet of Things); (3) elicitation, archiving and processing of very large data sets with the application of big data techniques; (4) various techniques of machine learning; and (5) advanced forms of human-computer interaction. Medical schools, therefore, need to integrate these technologies into their medical curriculum to meet digital transformation and innovation requirements.

In addition to health science and health technology, two more skill areas were identified in this study, namely product development & design and marketing & entrepreneurship. These non-technical skills are also important for health technology developers. Design thinking, a methodology for creative problem solving, was ranked as the most important skill in the product development & design category. Numerous studies have highlighted the significance of design thinking in healthcare, including those by Altman et al. (36), Valentine et al. (37), Przybilla et al. (38) and Compton-Phillips & Namita (39) among many others. According to Johansson-Sköldberg et al. (40), design thinking comprises several attributes and meanings. These include design thinking as the creation of artefacts, as a reflexive practice, as a problem-solving activity, as a way of reasoning/making sense of things, and as creation of meaning. Despite this variety, all share the central skill which is the instillation of user demand and experiences into the process of product design. Design thinking is a systematic innovation process that prioritizes deep empathy for end-user desires, needs and challenges to fully understand a problem in hopes of developing more comprehensive and effective solutions (41). This core concept of design thinking is very important and relevant to healthcare. Different groups of health technology end-users, especially patients, have different features and therefore require different attention.

Our results demonstrated three areas required for development in order to boost the health technology industry. These included (1) the establishment of national standards, (2) a market for health technology and investment for sustainable growth; and (3) ecosystem for the medical device industry that includes technology and human resources. These data are aligned with the Industry Foresight Project of the Office of Industrial Economics, Ministry of Industry of Thailand (42). Lack of national standards and its infrastructures, such as a national center for health technology certification, is the most important issue which requires urgent attention. Since the health technology development process requires vigorous monitoring, its process and products should be certified by an autonomous agency. Adoption of international standards are unavoidable if the products are to be distributed to other countries. Therefore, understanding the device certification concept and process are important for all stakeholders in the device development industry.

Our qualitative study underscores the transformative role of technology in healthcare and identifies four critical skill domains for health technology developers: Health Science, Health Technology, Product Development & Design, and Marketing & Entrepreneurship. Many different sectors will be affected significantly by these findings. They draw attention to the necessity of interdisciplinary curricula that incorporate these various skill sets and may even lead to the creation of new academic disciplines from an educational standpoint. Our findings which point to the necessity of workforce upskilling and collaborations with technology sectors highlight the significance of encouraging innovation and technological adaptation for the healthcare sector. In order to strike a balance between innovation, patient safety and data privacy, legislators and regulators may need to create more flexible frameworks. In terms of the economy, the addition of marketing and entrepreneurship skills indicates possible expansion prospects in the health technology industry which may spur innovation and employment development.

While our findings point to a shift toward more patient-centered healthcare, the emphasis on user experience and personalized medicine also raises concerns about health equity and access. These developments may lead to better health outcomes. In the context of global health, these abilities may be essential for tackling global health issues but their suitability in various settings, especially in low- and middle-income nations, must be taken into account. Strict attention must be paid to matters like data privacy, algorithmic bias and the possible aggravation of health disparities as ethical considerations are highlighted by the inclusion of technology and ethics as a required skill. In summary, our research points to a future in which healthcare will become more interdisciplinary, personalized and technology-driven. Coordinated efforts between the public health, business, education and policy sectors will be necessary to fully realize the potential of these advancements. Subsequent investigations ought to concentrate on the proficient application of these discoveries in diverse settings and the assessment of their enduring influence on healthcare systems and results.

Our findings about the skills needed by health technology developers may not be applicable or generalizable due to a variety of reasons such as geographic restrictions, bias in expert selection, temporal relevance, cultural context, market maturity, the breadth of health technologies and the interdisciplinary nature of the field. We employed a number of tactics to deal with these potential problems. In order to capture a wide range of perspectives we used open-ended questions in the initial interviews and the iterative Delphi method to balance individual biases. Additionally, we made sure the panel of experts was diverse and had experience in multiple sectors and countries. We emphasized core competencies essential to health technology development that ought to be transferable across various cultural contexts and concentrated on foundational skills likely to remain relevant despite technological changes. To ensure a balanced viewpoint, we also included experts with knowledge of both mature and emerging markets.

We suggest that in order to improve the transferability and generalizability of our findings, scholars and professionals should contextualize the findings to their own local settings, taking into account differing cultural considerations, technological infrastructure and regional healthcare systems. It is possible to compare and improve results by using our methodology as a framework for conducting comparable studies in various settings. Periodical reevaluation of the identified skills is also necessary in light of new technological advancements and evolving healthcare demands. Moreover, it will be critical to take into consideration the interactions among the recognized skill domains and how they might appear differently in distinct health technology subfields. We acknowledge that more research is necessary to validate and build upon our findings in various contexts, even though we have made every effort to provide results that are as transferable and generalizable as possible within the limitations of our study design. With continued use, this method will help to create a more thorough understanding of the competencies needed by health technology developers in various contexts.

## Limitations

Although our study identified the skills needed for health technology developers, there were some limitations. First, the identified skill domains are admittedly broad and may not capture the specialized competencies required for specific health technology applications. For instance, developing a computerized prescriber order entry system requires different technical skills (clinical workflow analysis, medication databases, alert algorithms) compared to designing pharmacogenomic prediction systems (bioinformatics, statistical modeling, genomic data interpretation). The generalized framework, while useful for foundational competency planning, may require subspecialization for specific technology domains.

Second, the limited emphasis on regulatory skills in our findings—despite regulatory approval being critical for health technology commercialization—raises questions about the comprehensiveness of stakeholder input. This may reflect a limitation of the Delphi method itself: regulatory requirements are mandatory rather than preference-based, and their prioritization should be independent of stakeholder consensus. Future research should include regulatory experts as distinct participants and separately assess mandatory vs. preference-based competencies to ensure comprehensive skill identification for health technology developers.

Another significant limitation of this study is the exclusive focus on high-level stakeholders while excluding frontline healthcare professionals (e.g., physicians, nurses, health IT workers) who are primary end-users of health technologies. Frontline professionals possess critical insights into practical implementation challenges, usability requirements, and clinical workflow integration that may differ substantially from administrative perspectives. This exclusion may have resulted in emphasis on strategic and business-oriented skills rather than user-centered technical competencies. The identified skill domains therefore represent a “top-down” administrative perspective rather than a comprehensive view including end-user insights. Future research should incorporate frontline healthcare professionals to capture essential competencies from both strategic and practical implementation perspectives.

In addition, this study relies entirely on qualitative methods and expert opinions without quantitative validation of actual skill demand in the health technology workforce. The absence of large-scale surveys, job market analysis, or statistical modeling represents a significant limitation. Future research should incorporate quantitative validation through: (1) surveys of health technology professionals to validate identified skill domains, (2) analysis of job postings to determine actual market demand for specific competencies, (3) content analysis of industry certification requirements, and (4) statistical modeling examining relationships between identified skills and professional success metrics. Such quantitative validation would transform these expert-derived insights into empirically-supported competency frameworks suitable for curriculum development and training programs.

This study identifies critical skill domains. However, it does not address institutional barriers that may impede implementation in Thailand's educational landscape (e.g., limited funding for health technology education, outdated curricula inadequately integrating emerging technologies, insufficient industry-academia collaboration, lack of specialized faculty). The absence of a national health technology certification center (identified by participants) creates additional disconnects between educational outcomes and industry requirements. Successful implementation requires coordinated efforts including industry-academia partnerships, dedicated funding streams, and national competency certification standards. Future research should systematically examine these implementation barriers and propose strategies for developing Thailand's health technology workforce.

The fast-changing nature of AI, particularly generative AI and machine learning applications in healthcare, suggests that skill requirements will continuously evolve beyond the identified framework. Emerging areas such as AI ethics, explainable algorithms, and human-AI collaboration may become critical competencies not fully addressed in current domains. Additionally, technologies like quantum computing in drug discovery and advanced predictive analytics will likely require specialized skills that transcend traditional boundaries between technical and clinical expertise. Future studies should incorporate dynamic technology assessments to ensure skill frameworks remain relevant in the rapidly evolving digital health landscape.

Although this study achieved statistically significant consensus (Kendall's *W* > 0.5, *p* < 0.01) after three Delphi rounds, the predetermined stopping point may have limited consensus refinement. Some studies continue until consensus coefficients exceed 0.7–0.8 or until response stability is achieved, suggesting additional rounds might have yielded stronger agreement and resolved persistent ranking variations between stakeholder groups. Future studies should consider adaptive stopping criteria based on consensus strength rather than predetermined round limits to maximize expert input.

Finally, critical issues (e.g., patient data privacy, algorithmic bias in AI systems, informed consent for digital health interventions, potential job displacement due to automation) require deeper exploration. The complexity of bioethics in digital health—including questions of data ownership, AI transparency, and equitable access to technology—suggests that ethical competencies may require more detailed specification beyond general ethical awareness. Future research should examine specific ethical dilemmas in health technology development and identify concrete ethical competencies needed to address real-world challenges in digital health implementation.

## Conclusion

This qualitative study involving 16 healthcare stakeholders in Thailand successfully identified four essential skill domains required for health technology developers: (1) Health Science, (2) Health Technology, (3) Product Development & Design, and (4) Marketing & Entrepreneurship. Through three rounds of Delphi consensus building, scientific method and artificial intelligence for healthcare emerged as the highest-priority skills in their respective domains, while design thinking and digital healthcare marketing were prioritized in product development and business domains. The study revealed that health technology development requires interdisciplinary competencies spanning clinical knowledge, technical expertise, user-centered design capabilities, and business acumen. These findings provide a foundational framework for curriculum development in health technology education programs and professional training initiatives. However, the generalizability of these findings beyond Thailand's healthcare context requires validation through broader international studies that include frontline healthcare professionals and quantitative validation methods. A systematic approach to developing these identified competencies represents a critical success factor for preparing human capital in technology-driven healthcare systems.

## Data Availability

The original contributions presented in the study are included in the article/Supplementary Material, further inquiries can be directed to the corresponding author.

## References

[1] Yeung AW Torkamani A Butte AJ Glicksberg BS Schuller B Rodriguez B The promise of digital healthcare technologies. Front Public Health. (2023) 11:1196596. 10.3389/fpubh.2023.1196596 37822534 PMC10562722

[2] Mumtaz H Riaz MH Wajid H Saqib M Zeeshan MH Khan SE Current challenges and potential solutions to the use of digital health technologies in evidence generation: a narrative review. Front Dig Health. (2023) 5:1203945. 10.3389/fdgth.2023.1203945 PMC10568450 37840685

[3] Dahlman KB Weinger MB Lomis KD Nanney L Osheroff N Moore DE Integrating foundational sciences in a clinical context in the post-clerkship curriculum. Med Sci Educ. (2018) 28(1):145–54. 10.1007/s40670-017-0522-1 29632705 PMC5889049

[4] de Bruin ABH Schmidt HG Rikers RMJP. The role of basic science knowledge and clinical knowledge in diagnostic reasoning: a structural equation modeling approach. Acad Med. (2005) 80(8):765–73. 10.1097/00001888-200508000-00014 16043534

[5] Finnerty EP Chauvin S Bonaminio G Andrews M Carroll RG Pangaro LN. Flexner revisited: the role and value of the basic sciences in medical education. Acad Med. (2010) 85(2):349–55. 10.1097/ACM.0b013e3181c88b09 20107367

[6] Thimbleby H. Technology and the future of healthcare. J Public Health Res. (2013) 2(3):28. 10.4081/jphr.2013.e28 PMC4147743 25170499

[7] Vatandsoost M Litkouhi S. The future of healthcare facilities: how technology and medical advances may shape hospitals of the future. Hosp Pract Res. (2019) 4(1):1–11. 10.15171/hpr.2019.01

[8] World Health Assembly, 60. Health technologies. World Health Organization (2007). Available online at: https://apps.who.int/iris/handle/10665/22609 (Accessed July 28, 2024).

[9] Chehri A Mouftah HT. Internet of things—integrated IR-UWB technology for healthcare applications. Concurr Comput Pract Exp. (2019) 32(2):e5454. 10.1002/cpe.5454

[10] Sox H Stern S Owens D Abrams HL. *Assessment of Diagnostic Technology in Health Care: Rationale, Methods, Problems, and Directions: Monograph of the Council on Health Care Technology*. Washington, DC: National Academies Press (US) (2014). 25144061

[11] Chui K Alhalabi W Pang S Pablos P Liu R Zhao M. Disease diagnosis in smart healthcare: innovation, technologies and applications. Sustainability. (2017) 9(12):2309. 10.3390/su9122309

[12] Gonzalo JD Dekhtyar M Starr SR Borkan J Brunett P Fancher T Health systems science curricula in undergraduate medical education. Acad Med. (2017) 92(1):123–31. 10.1097/ACM.0000000000001177 27049541

[13] Rowe RJ Bahner I Belovich AN Bonaminio G Brenneman A Brooks WS Evolution and revolution in medical education: health system sciences (HSS). Med Sci Educ. (2020) 31(1):291–6. 10.1007/s40670-020-01166-x 33224556 PMC7668405

[14] Abernethy A Adams L Barrett M Bechtel C Brennan P Butte A The promise of digital health: then, now, and the future. NAM Perspect. (2022) 6(22):2–8. 10.31478/202206e PMC9499383 36177208

[15] Aung YYM Wong DCS Ting DSW. The promise of artificial intelligence: a review of the opportunities and challenges of artificial intelligence in healthcare. Br Med Bull. (2021) 139(1):4–15. 10.1093/bmb/ldab016 34405854

[16] Xu A Johari SA Khademolomoom AH Khabaz MT Umurzoqovich RS Hosseini S Investigation of management of international education considering sustainable medical tourism and entrepreneurship. Heliyon. (2023) 9(1):e12691. 10.1016/j.heliyon.2022.e12691 36685466 PMC9853308

[17] Joint Commission International. Available online at: https://www.jointcommissioninternational.org (Accessed July 22, 2023).

[18] Medical Tourism Association. Medical Tourism Index 2020–2021. Thailand | Global Healthcare Destination. Available online at: https://www.medicaltourism.com/destinations/thailand (Accessed July 25, 2023).

[19] DiCicco-Bloom B Crabtree BF. The qualitative research interview. Med Educ. (2006) 40(4):314–21. 10.1111/j.1365-2929.2006.02418.x 16573666

[20] Akins RB Tolson H Cole BR. Stability of response characteristics of a delphi panel: application of bootstrap data expansion. BMC Med Res Methodol. (2005) 5(1):1–12. 10.1186/1471-2288-5-37 16321161 PMC1318466

[21] Williams PL Webb C. The Delphi technique: a methodological discussion. J Adv Nurs. (1994) 19(1):180–6. 10.1111/j.1365-2648.1994.tb01066.x 8138622

[22] Niederberger M Spranger J. Delphi technique in health sciences: a map. Front Public Health. (2020) 8:457. 10.3389/fpubh.2020.00457 33072683 PMC7536299

[23] Holey EA Feeley JL Dixon J Whittaker VJ. An exploration of the use of simple statistics to measure consensus and stability in delphi studies. BMC Med Res Methodol. (2007) 7(1):1–10. 10.1186/1471-2288-7-52 18045508 PMC2216026

[24] Liu W Hu M Chen W. Identifying the service capability of long-term care facilities in China: an e-Delphi study. Front Public Health. (2022) 10:884514. 10.3389/fpubh.2022.884514 35844860 PMC9277176

[25] Mohd Noor N Rasli A Abdul Rashid MA Mubarak MF Abas IH. Ranking of corporate governance dimensions: a Delphi study. Adm Sci. (2022) 12(4):173. 10.3390/admsci12040173

[26] Shi Y Sun S Deng J Liu S Yin T Peng Q Establishment and application of an index system for the risk of drug shortages in China: based on Delphi method and analytic hierarchy process. Int J Health Policy Manag. (2022) 11(12):2860–8. 10.34172/ijhpm.2022.6360 35297233 PMC10105199

[27] Watson S. The scientific method: is it still useful? Faculty publications and presentations (2004). Available online at: https://digitalcommons.liberty.edu/educ_fac_pubs/11 (Accessed July 22, 2023).

[28] Nichols AJ Stephens AH. The scientific method and the creative process: implications for the K-6 classroom. J Learn Through Arts. (2013) 9(1):2–6. 10.21977/D99112599

[29] Moraes SD. Scientific method and research in health: orientation for professional practice. J Hum Growth Dev. (2019) 29(1):5–9. 10.7322/jhgd.157742

[30] Thomas JW. A review of research on project-based learning. (2000). Available online at: http://www.bie.org/research/study/review_of_project_based_learning_2000 (Accessed July 22, 2023).

[31] Johnson KB Wei W Weeraratne D Frisse ME Misulis K Rhee K Precision medicine, AI, and the future of personalized health care. Clin Transl Sci. (2020) 14(1):86–93. 10.1111/cts.12884 32961010 PMC7877825

[32] Vogt C Gersch M Spies C Bengler K. Digital transformation in healthcare: how the potential of digital health is tackled to transform the care process of intensive care patients across all healthcare sectors. In: Urbach N, Röglinger M, editors. *Digitalization Cases: How Organizations Rethink Their Business for the Digital Age*. Cham: Springer International Publishing Ag. (2019). p. 343–61.

[33] Belliger A Krieger DJ. The digital transformation of healthcare. In: North K, Maier R, Haas O, editors. Knowledge Management in Digital Change. Progress in IS. (2018). p. 311–26. Available online at: https://link.springer.com/chapter/10.1007%2F978-3-319-73546-7_19 (Accessed July 22, 2023).

[34] Faddis A. The digital transformation of healthcare technology management. Biomed Instrum Technol. (2018) 52(s2):34–8. 10.2345/0899-8205-52.s2.34 29775387

[35] Pousttchi K Gleiss A Buzzi B Kohlhagen M. Technology impact types for digital transformation. IEEE 21st Conference on Business Informatics (CBI) (2019). p. 487–94. 10.1109/CBI.2019.00063

[36] Altman M Huang TTK Breland JY. Design thinking in health care. Prev Chronic Dis. (2018) 15(15):E117. 10.5888/pcd15.180128 30264690 PMC6178900

[37] Valentine L Kroll T Bruce F Lim C Mountain R. Design thinking for social innovation in health care. Des J. (2017) 20(6):755–74. 10.1080/14606925.2017.1372926

[38] Przybilla L Klinker K Wiesche M Krcmar H. A human-centric approach to digital innovation projects in health care: learnings from applying design thinking. PACIS 2018 Proceedings (2018). p. 226

[39] Compton-Phillips A Mohta NS. Care redesign survey: how design thinking can transform health care. NEJM Catalyst. (2018) 4(3):4–6. 10.1056/CAT.18.0159

[40] Johansson-Sköldberg U Woodilla J Çetinkaya M. Design thinking: past, present and possible futures. Creativity and innovation management. Creat Innov Manage. (2013) 22(2):121–46. 10.1111/caim.12023

[41] Roberts JP Fisher TR Trowbridge MJ Bent C. A design thinking framework for healthcare management and innovation. Healthcare. (2016) 4(1):11–4. 10.1016/j.hjdsi.2015.12.002 27001093

[42] OIE. Industry foresight project of the office of industrial economics, Ministry of Industry of Thailand. Available online at: https://www.oie.go.th/assets/portals/1/files/study_report/industryforesight2020.pdf (Accessed July 25, 2023).

