# Supplementary material for: Stakeholder perspectives on skills required for health technology developers: a qualitative study in Thailand
Source: Front Digit Health. 2025 Jul 18;7:1578782. doi: 10.3389/fdgth.2025.1578782 (PMC12313557; doi:10.3389/fdgth.2025.1578782)
Supplement: Supplementary file 1 [file Datasheet1.docx]

**APPENDIX**

**Appendix A. Delphi Approach**

We employed a modified e-Delphi technique, which is a structured communication method using online questionnaires over multiple rounds to achieve consensus among experts.

1. **Type**: Modified e-Delphi (online implementation of the classic Delphi technique)
2. **Sampling Criteria**: Same as the initial interview phase, with all 16 interviewees invited to participate in the Delphi rounds.
3. **Reliability and Validity**:

- Content Validity: The initial questionnaire was based on the qualitative analysis of interview data and was reviewed by three external experts for relevance and comprehensiveness.
- Face Validity: A pilot test of the questionnaire was conducted with five non-participant experts to ensure clarity and ease of use.
- Test-Retest Reliability: We calculated intraclass correlation coefficients between rounds to assess the stability of responses.

1. **Analysis Approach**:

- Quantitative: Kendall's W coefficient of concordance was used to measure the degree of agreement among experts.
- Qualitative: Thematic analysis of open-ended comments provided in each round.

1. **Consensus Definition**: We defined consensus as a Kendall's W ≥ 0.5 for each skill domain, with statistical significance (p < 0.01).
2. **Rounds**:

- Round 1: Experts ranked skills within each domain identified from the interviews.
- Round 2: Experts reviewed aggregated results from Round 1 and re-ranked skills.
- Round 3: Final ranking and comments on the consolidated list of skills.

1. **Stopping Criteria**: The Delphi process was stopped when either consensus was reached (as defined above) or after three rounds, whichever came first.
